# Developing Community Resources for Nucleic Acid Structures

**DOI:** 10.3390/life12040540

**Published:** 2022-04-06

**Authors:** Helen M. Berman, Catherine L. Lawson, Bohdan Schneider

**Affiliations:** 1 Department of Chemistry and Chemical Biology, Rutgers, The State University of New Jersey, Piscataway, NJ 08854, USA; berman@rcsb.rutgers.edu; 2 Institute for Quantitative Biomedicine, Rutgers, The State University of New Jersey, Piscataway, NJ 08854, USA; cathy.lawson@rutgers.edu; 3 Institute of Biotechnology of the Czech Academy of Sciences, 252 50 Vestec, Czech Republic

**Keywords:** nucleic acid structures, nucleic acid conformation, biological structure database, DNA, RNA, validation standards

## Abstract

In this review, we describe the creation of the Nucleic Acid Database (NDB) at Rutgers University and how it became a testbed for the current infrastructure of the RCSB Protein Data Bank. We describe some of the special features of the NDB and how it has been used to enable research. Plans for the next phase as the Nucleic Acid Knowledgebase (NAKB) are summarized.

## 1. Introduction

The first single crystal structures of nucleic acids were determined in the 1970s, almost twenty years after the model of the DNA double helix based on fiber data was published [1,2]. Short fragments of RNA yielded the first atomic-level views of the double helix and demonstrated conformational flexibility [3,4,5]. These structures were archived as small molecules in the Cambridge Crystallographic Database (CSD) [6]. The structure of tRNA, determined in 1974 [7,8,9], showed that RNA can fold into a compact structure and demonstrated the importance of tertiary interactions. As DNA synthesis became possible, structures of the DNA double helix with predefined sequences were determined. The first structures were left-handed Z-form DNA fragments [10], and in 1981, the first single crystal structure of a full turn of B-form DNA was published [11]. The tRNA structures and larger nucleic acid fragments were archived in the Protein Data Bank (PDB [12]). By 1990, there were nearly 100 publicly released nucleic acid structures, thus allowing analyses of sequence-dependent features, hydration patterns, and ligand interactions.

During the late 1970s and 1980s, several faculty members in the Chemistry Department at Rutgers University focused their research on nucleic acids. Ken Breslauer worked on the macroscopic properties of nucleic acids using calorimetric approaches [13,14,15,16]; these works, seminal for the understanding of thermodynamics of DNA, have continued to this day [17,18,19,20]. Roger Jones developed new methods to synthesize DNA [21]. Jerry Manning developed the counterion condensation theory to understand DNA folding [22], and continued this work in collaboration with the Breslauer group [23]. Wilma Olson performed detailed analyses of the structure of DNA [24]. During that period, Helen Berman carried out nucleic acid crystallography research at the Institute for Cancer Research in Philadelphia and had close interactions with the Rutgers group. In 1989, she joined the Chemistry faculty at Rutgers.

The setting at Rutgers was ideal for collaborative studies using both experimental and computational approaches to investigate nucleic acid structure. It was necessary to have a resource that contained the structural information which resided in the CSD, in the PDB, or in the laboratories of individual researchers to facilitate these efforts. In collaboration with David Beveridge, with whom Berman was collaborating on computational analyses of nucleic acid hydration, Olson and Berman proposed to create the Nucleic Acid Database (NDB). In the early 1990s, funding was received from the National Science Foundation to establish “A Comprehensive Database of the Three-Dimensional Structures of Nucleic Acids”. The goal was to create a searchable database that would integrate information from several sources and make a variety of reports, thus enabling research on nucleic acid structure. 

## 2. Development of the Nucleic Acid Database

The first step in the development of the NDB was to collect and curate the structural data [25]. Coordinates were accessed from the CSD and the PDB. Each structure and experiment were carefully reviewed to create appropriate annotations beyond what was available from each resource. Rather than working directly with the flat files maintained by the PDB, the NDB imported the parsed data files into a relational database management system (DBMS). Sybase [26] was chosen as the DBMS in large part because it was being used by Genbank [27,28]. A query system called NDBquery was put into place. In the early years, distribution was accomplished via FTP and a system called Gopher [29]. By 1995, a web server was set up, which generated a modest amount of activity to access and analyze the 350 structures represented in the NDB. The NDB was actively involved in the development of mmCIF, whose data model is compatible with a relational DBMS. By 1996, mmCIF [30] became the master format for the NDB. The software that was developed and the experience gained using this data representation set the stage for the management of the Protein Data Bank using mmCIF as the master format by the Research Collaboratory for Structural Bioinformatics (RCSB) beginning in 1998.

The NDB also became a driver for the creation of geometrical standards for nucleic acid structures. Careful analysis of high-resolution structures from CSD permitted the calculation of standard reference bond distances and angles for the bases, sugars, and phosphates of nucleic acids [31,32]. Using these values, Parkinson et al. [33] created new parameters that enabled improved refinement of nucleic acid-containing crystal structures against their experimental data. Those standards were widely used. In 1998, the NDB helped organize a conference whose outcome was the standard coordinate frame definition for nucleic acid bases [34]. This standard became widely adopted by researchers studying nucleic acid base morphology.

## 3. Features of the NDB

In addition to facilitating access to primary data for nucleic acid structures, the NDB provides tables of derived features, such as classifications of base pairing topologies [35], backbone torsion angles, and conformational and base pair classifications [36,37]. 

**Figure 1:**
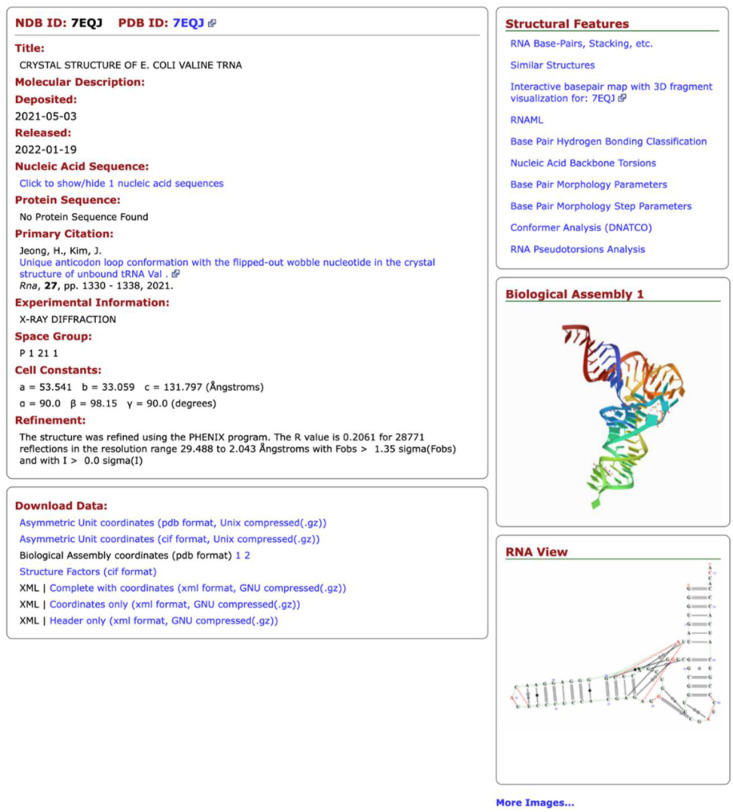
NDB Atlas page of a tRNA structure.

The NDB also offers different types of data visualization and presentation. The most important is the NDB Atlas page (Figure 1), which gives summary information about the structure, visualizations of the crystal asymmetric unit, the biological unit, unit cells, and for RNA structures; it provides a view that combines the secondary and tertiary structural features. Links to other resources are also provided.

The functionality of the NDB and its query engine was first and foremost driven by research projects on the nucleic acid structural and computational biologists. Careful attention was given to the quality and uniformity of the metadata so that it would be possible to use Boolean logic to create queries; individual questions could be made into logical constructs joined by logical AND, OR, and NOT. This requirement represented a challenge for building a robust system of precisely defined terms incorporated into a formal computer-readable language; mmCIF was that dictionary.

**Figure 2:**
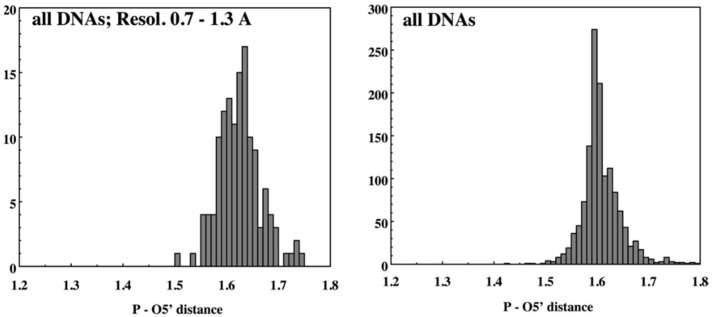
Example NDB report of geometric features of nucleic acids, based on structures available in the 1990s. Histograms show the P–O5′ valence distances in high-resolution DNA, and in all DNA structures.

**Figure 3:**
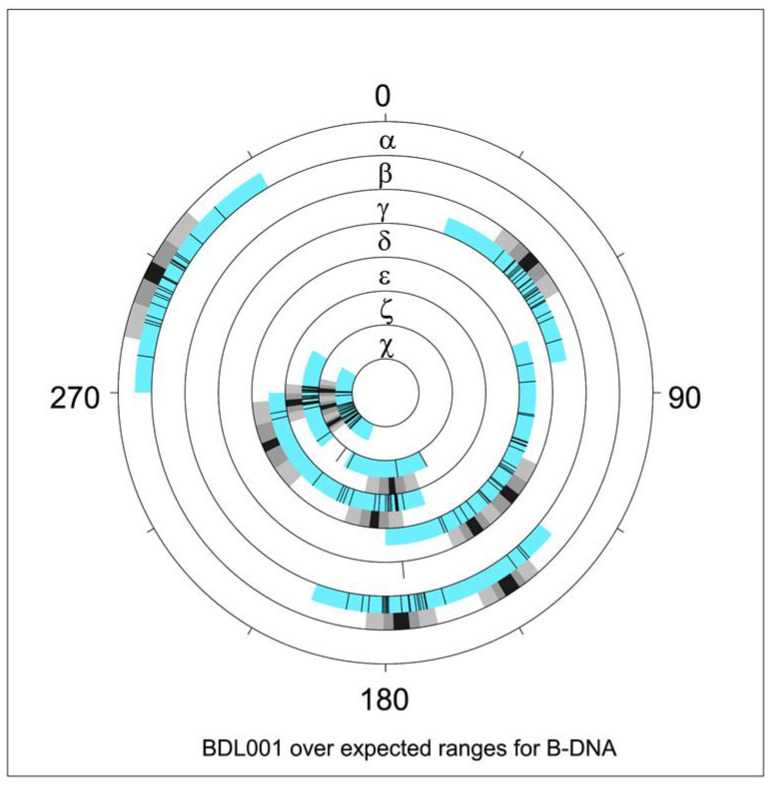
NDB graphical report of torsion angle distribution [38] for the Drew–Dickerson dodecamer, PDB ID 1BNA, NDB ID BDL001 [11]. Blue sectors indicate torsion angle limits for all structures annotated as B–DNA. Overlaid black tick marks are measured torsion values for BDL001. Adjacent black/grey sectors denote average values and spreads of 1 and 2 estimated standard deviations. Note that two averages are indicated for several torsions, e.g., for δ (two distinct sugar puckers) and ε (BI versus BII forms). Values reflect NDB data available in 1996.

The NDB website was designed so that the user could select structures with features of interest and then use those structures for further analysis, e.g., through the creation of detailed tabular or graphical reports. Soon after the first functional version of the NDB was available, we started to use its potential to study the geometrical features of nucleic acids. The original NDB reporting capability allowed the user to obtain tabular reports of various properties of the selected nucleic acid structures from basic information about the publication or refinement parameters and graphical reports of selected geometric features such as bond distances (Figure 2) or torsion angles (Figure 3). Once funding for the NDB became limited in the 2000s, it was not possible to maintain these reporting capabilities.

## 4. Research Enabled by the NDB

**Figure 4:**
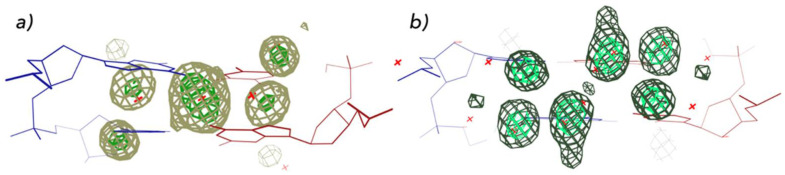
Sequence dependence of DNA hydration. Two distinct hydration patterns are shown in for the A-form major groove for (**a**) 5′–GC–3′ and (**b**) 5′–CG–3′, based on analyses of structures available in the mid–1990s NDB [44]. A more recent analysis of hydration using larger and functionally more relevant dinucleotide fragments is available at watlas.datmos.org/watna (accessed on 30 March 2022).

The NDB has been used by many researchers to analyze the structures of nucleic acids. There are over 1100 citations to the original NDB article. The type of research enabled by the NDB includes DNA conformational analyses [39], DNA structure prediction [40], RNA structure prediction [41], analyses of protein-nucleic acid interactions [42,43], and the creation of new specialty databases [44]. In our research, we have used the NDB to study a variety of aspects of nucleic acids. For example, we surveyed A, B, and Z-form double helical DNA structures and used Fourier averaging to determine hydration patterns, e.g., for DNA nitrogenous bases [45]. Both base and later phosphate studies showed sequence and conformation-dependent water position preferences (Figure 4). 

**Figure 5:**
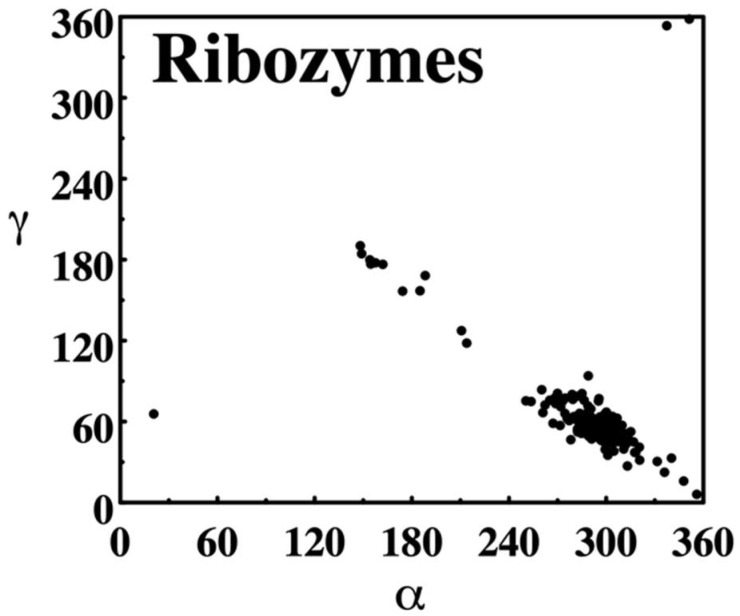
NDB torsion angle scatter plot. The distribution of backbone torsion angles α and ɣ observed in crystal structures of RNA annotated in the NDB as ribozymes in 1996 is shown. α describes rotations around the P–O5′ phosphodiester bond, ɣ around C5′–C4′ bond. Plots were created directly on the NDB website as PostScript formatted reports.

**Figure 6:**
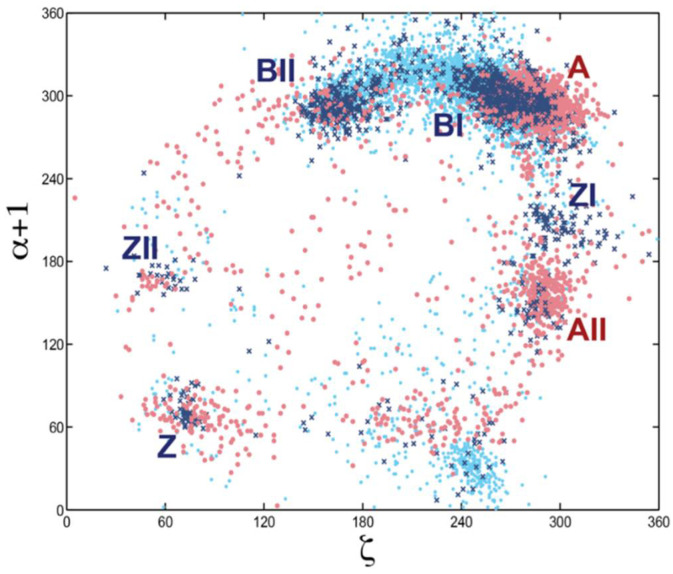
Scatter plot of backbone torsion angles ζ and α + 1. ζ shows the variation in rotation around the O3′–P phosphodiester bond and α + 1 around the P–O5′ bond (labeled α + 1 because this bond belongs to the sequentially following nucleotide). Data for the DNA alone is shown as dark blue crosses, for protein-DNA complexes: light blue dots, and for RNA: red dots. The scattergram shows data for all nucleotide residues in the 1998 NDB. Clusters of some major conformational types are labeled. This analysis revealed that no nucleic acid form can be unequivocally classified by torsion angle pairs; a more sophisticated multidimensional analysis was needed.

The growing volume of available crystal structures with ever growing sequence variability also led us to ask whether conformational properties of various DNA and RNA forms could be better characterized. This task posed new challenges to NDB querying and reporting capabilities. Specific subsets of structures were selected based on sequence, function, or structural features using SQL queries; their properties were reported as text or graphs (Figure 5). Ultimately, we were able to sharpen conformational definitions for established subtypes of A-B-Z forms (Figure 6) [46]. 

The growing number of nucleic acid structures and the appearance of new forms such as quadruplexes and large-folded RNAs demonstrated the plasticity of nucleic acid molecules. It became clear that the conformational space of nucleic acids is extremely complex and that capturing it would require a concerted understanding of base pairing motifs and the backbone structural variability. 

Early analyses showed that backbone conformational variability was fundamentally influenced by flexibility around the O3′–P–O5′ phosphodiester bonds that connected adjacent nucleotide residues, described by torsion angles ζ and α [47]. Our multidimensional statistical analysis, therefore, focused on dinucleotide fragments analyzed in torsion space, taking full advantage of the availability of the NDB and PDB. 

**Figure 7:**
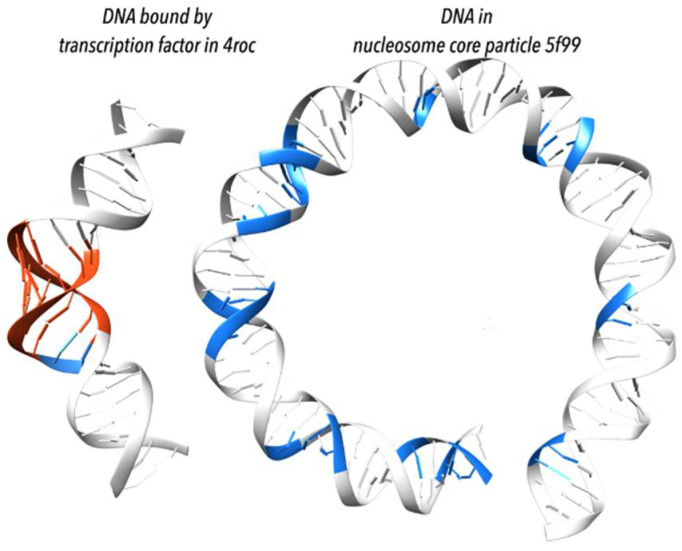
Transcription factors and proteins of the histone core particle bend DNA duplex differently [49]. (**Left**) bending by transcription factors is acquired mostly by local adaptation to the A form (highlighted in red); shown is DNA from complex with TFIIB–Related Factor Brf2 (PDB id 4ROC [51]). (**Right**) bending by the histone core particle is associated with the BII form (highlighted in blue); shown are first 75 base pairs from a histone core particle (PDB id 5F99 [52]); when statistically measured over many structures, the BII form appears in histone-wrapped DNA every tenth step corresponding to one full turn of duplex; the periodicity of the BII form appearance explains the DNA bending.

In the 2000s, research conducted by several groups concentrated on analysis of RNA backbone flexibility culminated in an RNA Consortium consensus set of dinucleotide conformers [48]. The effort was later complemented by an analogous set of DNA conformers [49] and, ultimately, a comprehensive classification system for dinucleotide fragments covering both DNA and RNA [50]. This classification algorithm provides an automated structural ranking of dinucleotide fragments at two levels of detail: fully geometrical classification into dinucleotide conformational classes (NtC) and a more human-accessible structural alphabet (CANA). The assignment of the CANA and NtC classes makes it possible to study the structural propensities of dinucleotide sequences. For example, analysis of DNA in transcription factors and in histone core particle complexes showed important trends of protein interactions with specific bending associated NtC classes (Figure 7) [49]. 

**Figure 8:**
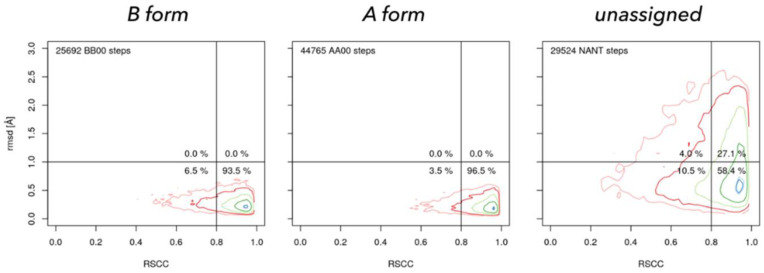
Geometrically well-defined dinucleotides fit well into their electron densities. Real Space Correlation Coefficient (RSCC, horizontal axis) measures how closely the model electron density resembles the experimental density and rmsd (vertical axis) measures how closely the geometry of the model resembles the closest NtC class in the so called golden set [50]. NtC class BB00 (**left**) characterizes the B form, AA00 (**center**) A form in both DNA and RNA, and NANT (**right**) are all unclassified dinucleotides. Geometrically unclassified dinucleotides fit significantly worse to the electron density.

NtC assignments have also inspired development of a new validation tool linking the global geometry criterion (closeness of fit to the nearest NtC class) and the quality of fit into electron density (Figure 8) [50]. It offers a simple information-rich graphical representation of the overall quality of nucleic acid structure in the form of a 2D graph.

**Figure 9:**
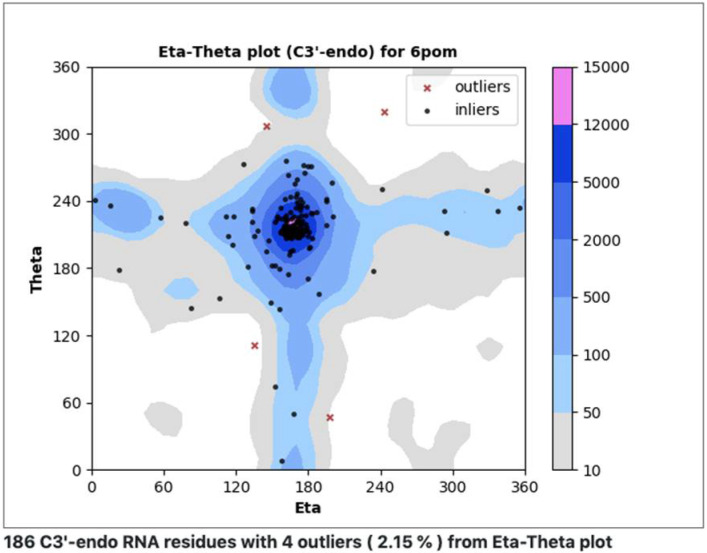
Pseudotorsion plot, a simple coarse-level RNA backbone conformation validation tool. Measured eta and theta (η,θ) pseudotorsion values for each trinucleotide (black dots, red x’s) are plotted against a quality-filtered virtual torsion angle distribution derived from a large number of RNA structures (contours), analogous to the Ramachandran plot for proteins.

In an additional effort to understand, classify, and validate nucleic acids, we have developed a procedure similar to Ramachandran analysis for proteins, making use of eta (η) and theta (θ) virtual torsion angles (pseudotorsions) [53,54]. Measured (η,θ) pairs define backbone conformations for each central residue within a trinucleotide. Plots are designed to quickly reveal rare conformations that may need extra checking (Figure 9). A web server was recently set up to investigate the utility of this approach for RNA structures determined using cryoEM (ptp.emdataresource.org) (accessed on 30 March 2022). 

## 5. Current State of Nucleic Acid Structural Biology

**Figure 10:**
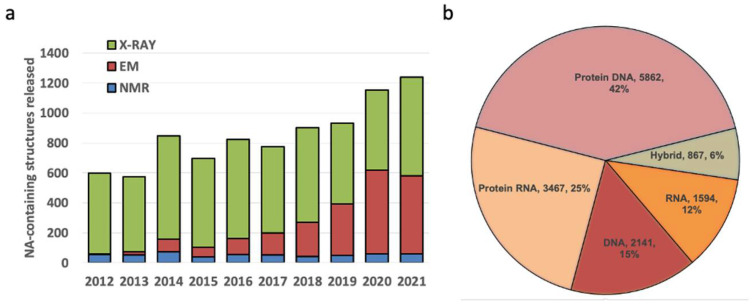
Current statistics for nucleic acid-containing structures. (**a**) New structures released into the PDB, by year and method; (**b**) Distribution of nucleic acid-containing structures. NDB archives and annotates structures determined using X-ray crystallography or NMR. Electron microscopy structures are not included in the NDB, but will be included in the NAKB, the planned successor to NDB.

**Figure 11:**
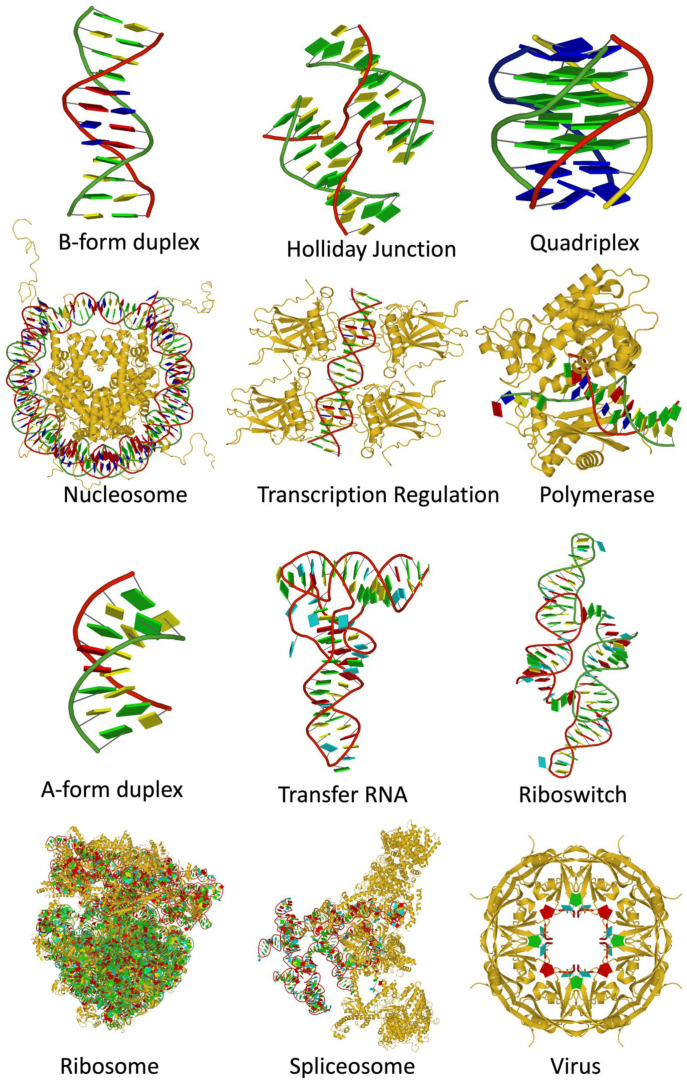
Diversity of structures containing DNA (top rows) and RNA (bottom rows). Nucleic acids are shown with ribbon backbones (random colors) and base blocks (A—red, C—yellow, G—green, T—blue, U—cyan). Proteins are shown as gold ribbons. From top left: B-form duplex DNA (1BNA [11]), Holliday junction (5DSB [55]), parallel-stranded DNA quadruplex (139D [56]), nucleosome core particle (1KX5 [57]), *trp* repressor/operator complex (1TRR [58]), DNA repair enzyme rev1 (6X6Z [59]), A-form duplex RNA (402D [60]), tRNA Asp (6UGG [61]), glutamine II riboswitch (6QN3 [62]), bacterial ribosome (4YBB [63]), spliceosomal E complex (6N7P [64]), Ebola virus matrix protein octamer (7K5L [65]). Images were generated using DSSR and PyMOL [66].

When the NDB was established in the early 1990s, most of the nucleic acid structures were small fragments with the exception of tRNA. There were a few structures of protein-nucleic acid complexes, limited to virus capsids with viral genomic RNA or DNA and transcription factors bound to duplex DNA. Molecular machines, such as the ribosome, were yet to be determined. In contrast, there are now more than 14,000 nucleic acid-containing structures in the PDB and NDB (Figure 10). A notable trend is the recent increase in the use of electron microscopy (EM) for structure determination. Protein/DNA complexes are the most abundant, followed by protein/RNA, DNA-only, and RNA-only. In addition to the increase in the number of structures, the structures are very diverse, as shown in Figure 11. 

These structures have significantly expanded our knowledge of structure/function relationships and raised the potential of new knowledge from systematic analyses of structure collections. Many different databases and tools have been created to enable specialized analyses of nucleic acid structures. Some have focused on DNA [67], some on RNA [68,69,70,71,72], and some on the interactions between proteins and nucleic acids [73,74]. A systematic long-term analysis of dinucleotides led to a unified RNA + DNA automated classification system [50] available at DNATCO (dnatco.datmos.org) (accessed on 30 March 2022). The NDB (ndbserver.rutgers.edu) (accessed on 30 March 2022) is unique in that all nucleic acid structures and their complexes are contained in a single resource.

## 6. Going Forward

The NDB is maintained to the extent that new structures and manually curated annotations are added each week, but there is little significant development since its last full funding in 2003. Even so, thousands of users from the Americas, Asia, Europe, and other locations continue to make multiple visits to the NDB website each month. The most heavily visited pages are Advanced Search and DNA and RNA galleries.

In 2018, the collaborative group of scientists managing both the NDB (at Rutgers) and RNAhub services (at Bowling Green State University) proposed to create the Nucleic Acid Knowledge Base (NAKB), with the goal of integrating information already in the NDB with additional sequence, structure, function, and interaction-based annotations for all major classes of NA-containing 3D structures. This new service, which will ultimately replace the NDB, is currently under construction. The NAKB aims to enable users to quickly find and download all structures and metadata relevant to their search topic, whether broad or focused, based on the NDB’s internal curation scheme, computationally generated annotations, and/or external database references for DNA, RNA, mixed NA, and for NA-binding enzymatic, regulatory, and structural proteins. All NA-containing structures in the PDB will be indexed, including structures obtained using Electron Microscopy. The NAKB will be updated weekly.

The NDB has employed manual expert curation collected over three decades to identify major NA secondary structure features (duplex, triplex, and quadruplex) and high-level classifications (e.g., ribosomal RNA or telomeric DNA), as well as interactions with ligands (e.g., minor groove binding) and protein classification [37]. Integrated computationally created annotations have included bond distance, angle, and torsion geometries, base and base-pair morphologies, as well as RNA 3D motifs, interactions (base pair types and parameters, base-to-backbone, and base stacking interactions), and RNA equivalence (3D structure similarity) classes [75,76].

New NAKB content will include equivalence class calculations for all nucleic acid molecule types (RNA, DNA, hybrid nucleic acids), enabling more accurate retrieval for closely related NA structures, analogous to the way that UniProt identifier mapping has improved search capabilities for related proteins in PDB [77]. Computationally derived annotations produced by DSSR software [78], including secondary structure features, sugar pucker type, and pseudo-torsion angles, will be added.

New search capabilities will be developed for specific classes of chemical modifications of nucleotides; nucleic acid 3D structure motifs by their common names, for example, G-Quadruplex, R-loop, Holliday Junction, Sarcin–Ricin, Kink-turn; ribosome functional states, e.g., full or single subunit, translational state, and numbers and positions of bound tRNAs; and deeper classification levels for selected proteins such as transcription factors.

The NAKB website will employ a modern web infrastructure with flexible data representation viewable on phones and tablets as well as desktop computers. For each NA-containing 3D structure, an atlas page will provide a summary overview of annotations as well as access to 1D, 2D, and 3D visualizations, external analysis tools, and file downloads. Mappings to external database links will initially include: PDB, Uniprot, RNACentral, Rfam. External analysis tools will include DNATCO and DNAproDB. Some of the reporting functions that were available in the original NDB so that the types of conformational analysis described earlier will be reenabled.
